# Guiding Needle‐Assisted Redistribution of Orbital Fat in Transconjunctival Lower Eyelid Blepharoplasty

**DOI:** 10.1111/jocd.70446

**Published:** 2025-09-12

**Authors:** Ke Wu, Jinfei Hou, Jialong Chen, Diandian Li, Jiaming Sun, Muran Zhou, Aimei Zhong

**Affiliations:** ^1^ Department of Plastic Surgery, Union Hospital, Tongji Medical College Huazhong University of Science and Technology Wuhan China; ^2^ Wuhan Clinical Research Center for Superficial Organ Reconstruction Wuhan China

**Keywords:** eyelid bags, lower blepharoplasty, tear trough deformity

## Abstract

**Background:**

Accurate fat redistribution after lacrimal trough ligament release by the conjunctival approach is difficult due to the limited surgical field of view. Therefore, creative methods are urgently needed.

**Aims:**

To explore an innovative technique focused on fat redistribution using a guiding needle in transconjunctival lower eyelid blepharoplasty to correct tear trough deformity.

**Methods:**

Twenty‐six patients underwent transconjunctival lower eyelid blepharoplasty. The retroseptal fat was dissected and laid out using a guiding needle for redistribution over the orbital rim. Closing sutures were knotted internally to the periosteum. Patients were followed for 6 months.

**Results:**

All patients diagnosed with predominant orbital fat bulge and tear trough deformity without noticeable lower eyelid skin laxity presented impressive correction of the eyelid bags and tear trough deformities at 6 months follow‐up. The average score for postoperative effect was 5.692 ± 0.618 on a scale of 1–6 (1 being poor and 6 being good).

**Conclusions:**

This guiding needle is a simple and effective tool for correcting eyelid bags and tear trough deformities by redistributing orbital fat in the transconjunctival lower eyelid blepharoplasty.

## 1 Introduction

Tear trough, commonly known as the lacrimal groove, is a distinct cutaneous groove that starts from the medial canthus and extends to the medial pupillary line. With aging, tear troughs become deeper and more visible as lower eyelid bags are bulgy [1, 2]. Anatomically, the tear trough is a natural anatomical depression located between the medial canthus and the infraorbital rim, extending medially from the medial pupillary line. It is formed by the attachment of the tear trough ligament, a fibrous band connecting the medial canthal tendon to the periosteum of the infraorbital rim, which provides structural support to the lower eyelid. The tear trough is closely associated with herniation of orbital fat through a weakened orbital septum. The tear trough is ligament‐supported and linked to orbital fat dynamics, whereas the nasojugal fold arises from soft tissue laxity. Clinically, the tear trough deformity is addressed through fat repositioning or grafting via transconjunctival approaches. To address this concern, various surgical approaches have been developed relying on lower eyelid blepharoplasty [3].

Conventional methods for correcting tear trough deformities involve using excess orbital septal fat to fill the anatomical defects in the tear trough area. Among these methods, the transconjunctival approach is popular in these surgeries due to its minimally invasive nature, quick recovery, and the absence of scar formation [4]. The frequently employed technique, initially suggested by Goldberg, involves the conservative excision of lateral fat pads. Subsequently, through an arcus marginalis transection, a subperiosteal dissection is conducted along the tear trough. This dissection serves to fill the tear trough by utilizing random‐pattern fat flaps from the medial and central pockets. Three to four externalized knots are required to maintain the fat pedicle in position [5]. Hidalgo later enhanced this technique by solely suturing the substantial portions of the medial and central fat pads, omitting the extensive pedicle fat pads. By integrating component techniques customized to address individual anatomical concerns and preserving the orbicularis muscle, this approach has proven to be effective and associated with a low incidence of complications and the need for revisions. Ultimately, orbital fat release successfully eliminates the tear trough deformity [6]. For closing, the suture knots are tied on the skin's surface, which exposes the suture material, requiring attentive medical care.

Due to the limited surgical field and technical difficulty associated with internal fixation of fat, coupled with the high recurrence rate of external fixation, Zhang et al. developed a technique utilizing syringe needle‐assisted internal fat fixation [7], while Han et al. employed barbed suture fixation [8]. However, the optimal position and angle for fat fixation vary according to individual facial anatomy. Furthermore, both syringe needles and barbed sutures carry risks of neurovascular injury, indicating a need for improved safety. To address these challenges, we have developed a set of guide needles for orbital fat fixation, aiming to enhance surgical efficiency while ensuring safety. This study introduces a technique that optimizes surgical outcomes in transconjunctival tear trough blepharoplasty through guided‐needle internal fat fixation, demonstrating procedural refinement in lower eyelid rejuvenation. Patient self‐assessment and surgery complications were recorded to assess the described method.

## 2 Materials and Methods

The study included the patients who underwent lower blepharoplasty at Wuhan Union Hospital, China, from January 2019 to September 2021. The inclusion criteria were as follows: (1) aged 18–55 years, male or female, (2) having bulging eye bags and tear trough deformity with an absence of significant skin laxity in the lower eyelids, (3) willingness to participate in the study and provide consent by signing a consent form, (4) willingness to undergo clinical follow‐up. On the other hand, the exclusion criteria were as follows: (1) diagnosis of severe mental abnormalities, psychological and personality disorders contraindications, and severe coagulopathy; (2) subject to previous reconstructive surgery with lower eyelid blepharoplasty or fat removal from the lower eyelid's orbital septum; (3) existence of contraindications to lower eyelid blepharoplasty; (4) patients with eye inflammation such as thyroid eye disease or idiopathic orbital inflammatory disease or conjunctivitis or infection of the skin around the eyes. Data for the following variables were collected: age, sex, concomitant procedures, follow‐up duration, complications, and self‐assessment reports.

### 2.1 Surgical Technique

Before the surgery, we marked the tear trough and the points for fixing the orbital fats. Additionally, entry points for the guiding needle were marked and positioned below the superficial projection of the marginal arc. To provide local anesthesia to the lower lid surgical area, we administered a mixture of 2% lidocaine (by mass) and adrenaline (1:200 000) via injection into the orbital septum through the inferior fornix of the conjunctiva and the tear trough region.

**FIGURE 1:**
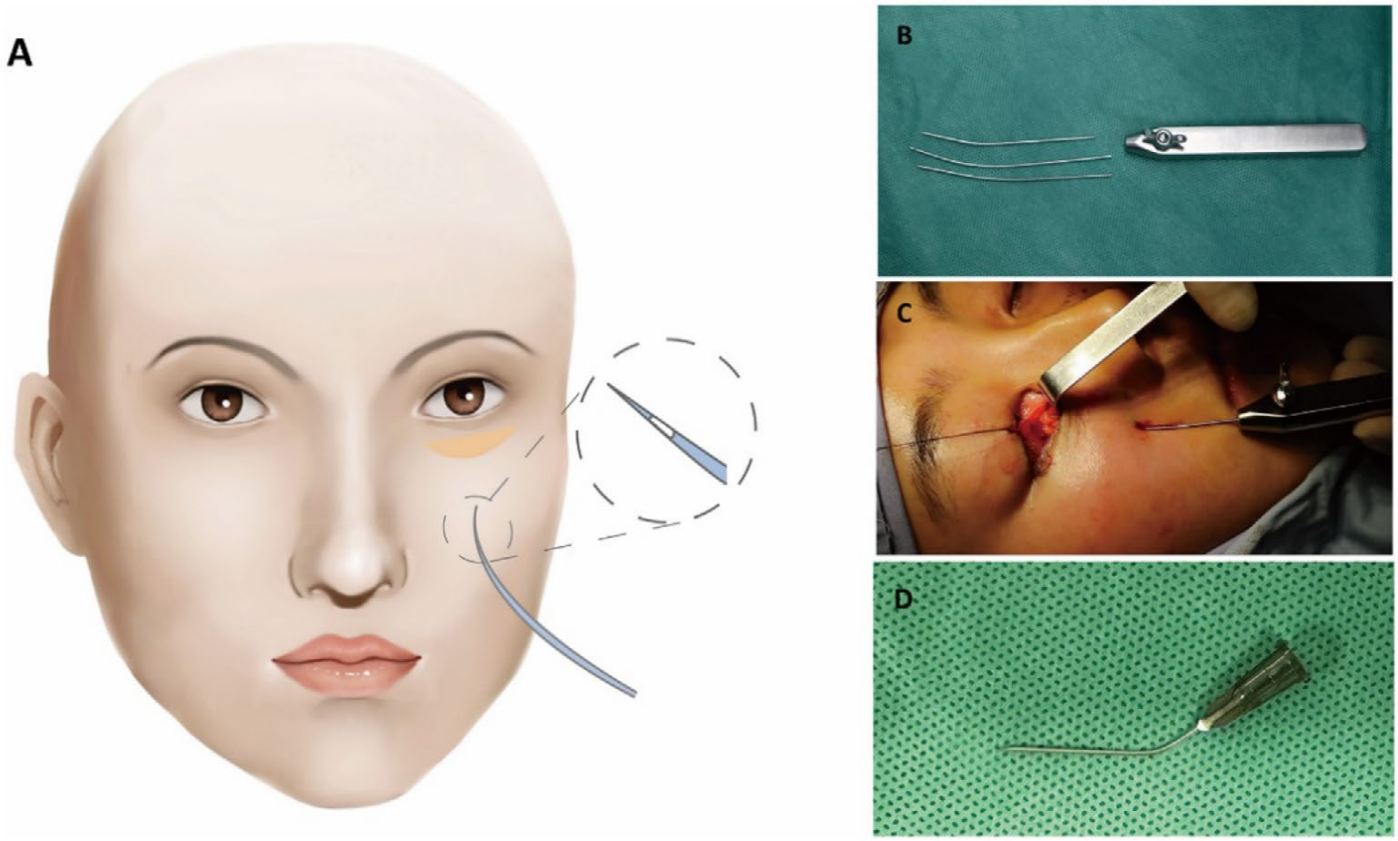
(A) Schematics illustrated the approximate position of the entry point for the needle on the face. (B) Guiding needle and needle holder. (C) Surgical process displaying the usage of the needle. (D) Modified syringe needle.

After local anesthesia, the lower eyelid conjunctiva was exposed and a 10–20 mm transconjunctival incision was made. From the conjunctival incision, the pre‐septal space was opened until the arcus marginalis was completely exposed. The premaxillary and prezygomatic spaces were dissected under direct vision. The orbital septum was then opened at a level close to the infraorbital rim to allow the handy release of the intraorbital fat, followed by partial excision of the medial, central, and lateral excess fat pads; the left bulging fat was then fixed at identified marked points to fill the tear trough. The guiding needle was equipped with a needle holder and then carefully inserted into the assigned entry point. After piercing through the soft tissue, the needle tip could be seen from the conjunctival incision site. Then, a suture was threaded into the hole of the guiding needle after fixing it to the excess septum fat. The guiding needle was then retreated to the subcutaneous level without detaching it from the skin; it was inserted again at an angle away from the previous insertion point through the subcutaneous soft tissue. The end of the suture was then taken away from the guiding needle and tied in a knot with the other end of the suture. Figure 1A shows the gross view of the guiding needle as well as its entry point in the orbital region during operation. Figure 1B,C shows images of the needles and operative process. Figure 1D shows a picture of the modified syringe needle.

**FIGURE 2:**
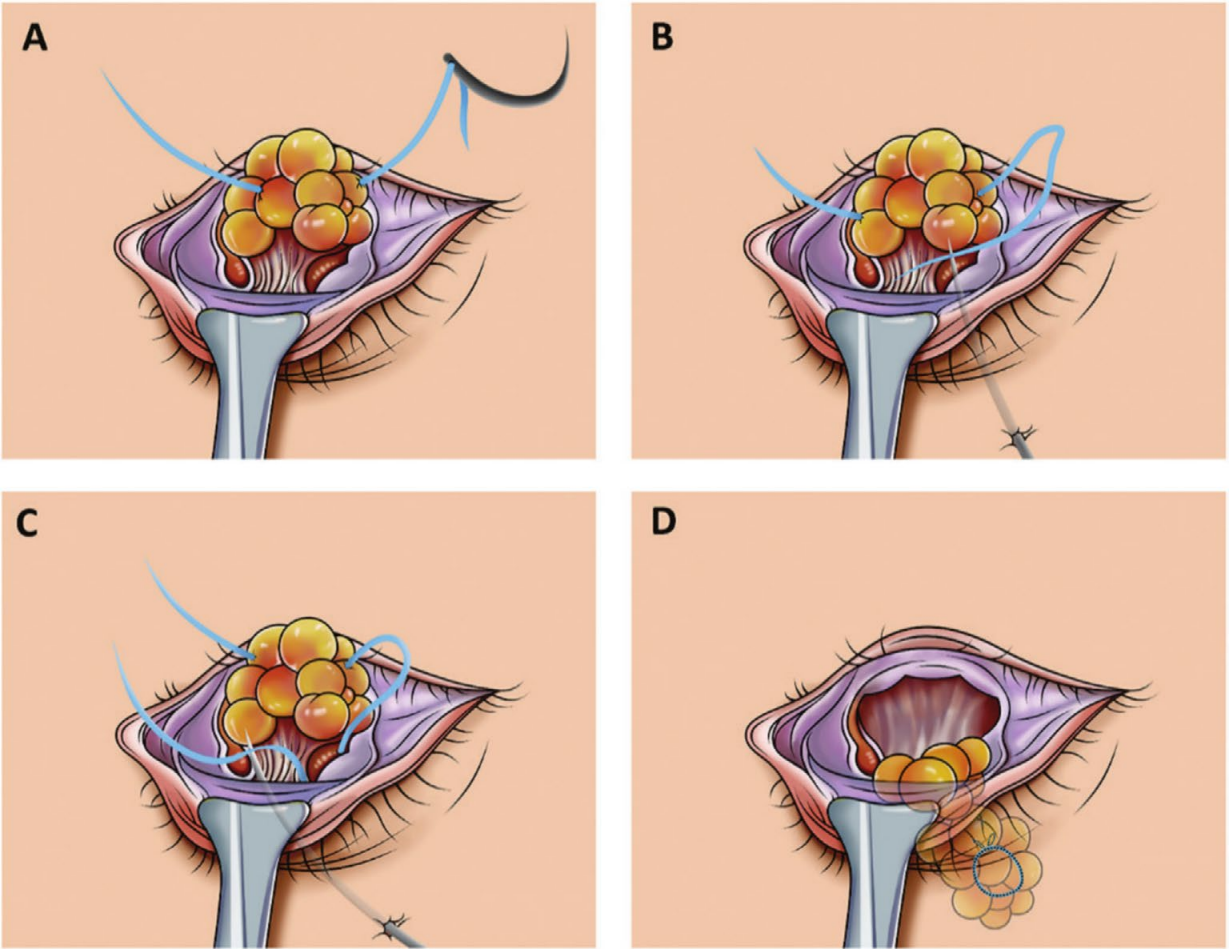
The process of transconjunctival lower eyelid blepharoplasty. (A) A normal needle with 6‐0 suture got pierced through the fat pad. (B) The suture was threaded to the hole of the guiding needle that was passed through the skin. (C) The needle with suture was retreated to the subcutaneous level without detaching it from the skin and inserted again at an angle away from the previous insertion point through the subcutaneous soft tissue. (D) The fat pad was tied in a knot with deep periosteum tissue around arcus marginalis.

**FIGURE 3:**
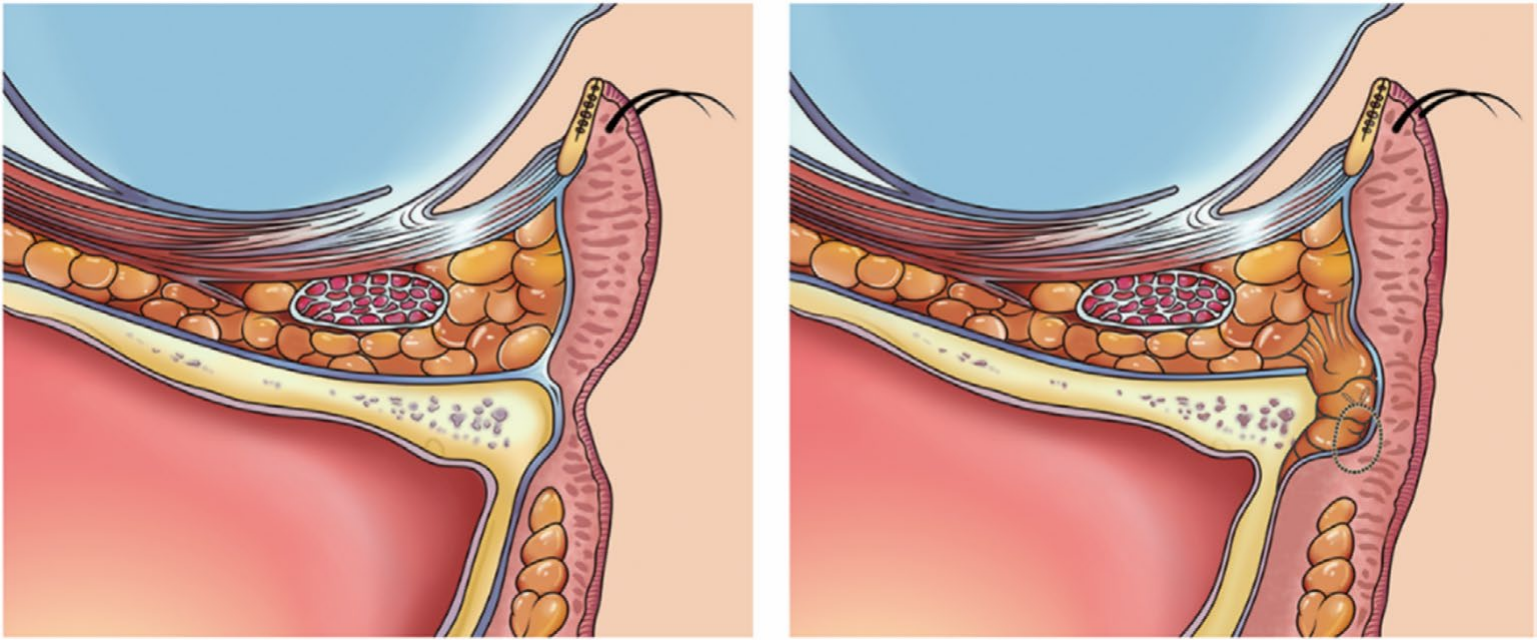
The sagittal view of the orbital region before (left) and post‐operation (right). The position of the subcutaneous knot could be observed.

The process above is illustrated in Figure 2. The same procedure was performed for fat transposition on the contralateral side. In cases of severe tear trough deformity requiring a larger amount of fat for correction, it may not be necessary to extract fat from the eye bags. Instead, all the available fat can be utilized to fill the tear trough. This approach involves applying the internal fixation technique with the guiding needle, allowing for the filling of the tear trough with orbital septal fat without the requirement for knots on the skin's surface. The sagittal plane before and after the operation and the position of the knot are illustrated in Figure 3. No sutures on the conjunctival incision were needed.

## 3 Results

### 3.1 Basic Patient Information

From January 2019 to September 2021, 26 cases of transconjunctival lower eyelid blepharoplasty utilizing the guiding needle for internal fixation of fat flaps were performed by a single plastic surgeon.

**FIGURE 4:**
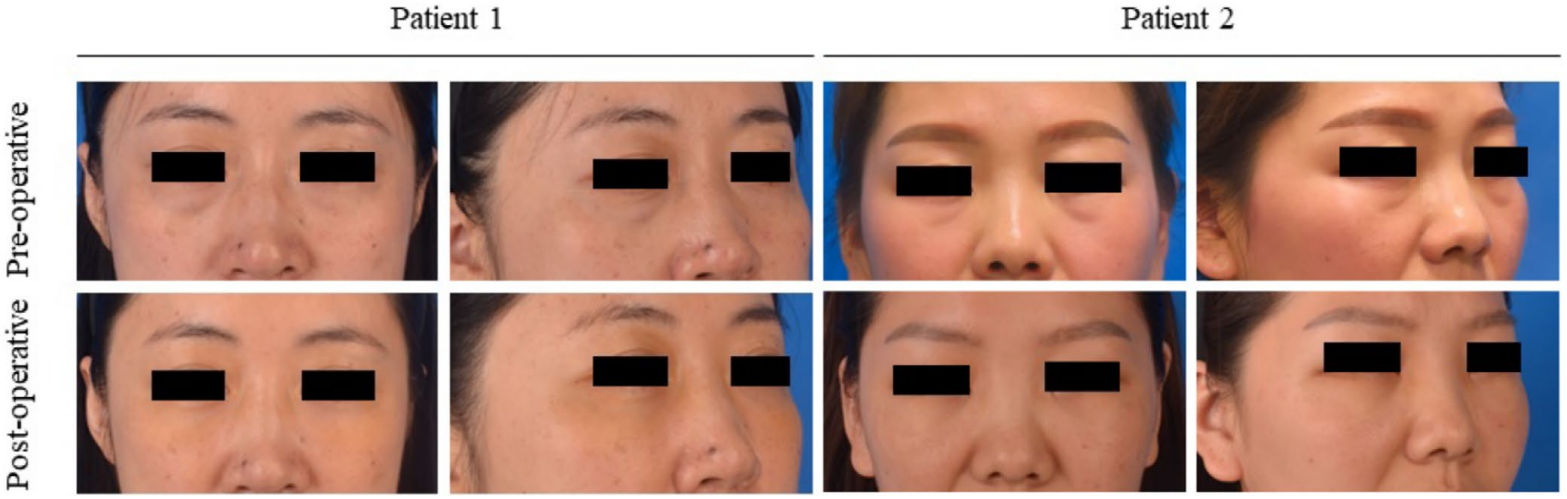
Clinical outcomes of guiding needle‐assisted redistribution of orbital fat in patients 1 and 2. Patient 1: A 34‐year‐old female patient received surgery with the Type I guide needle. Pre‐operative images and 6‐month post‐operative images. Patient 2: A 37‐year‐old female patient received surgery with the Type II guide needle. Pre‐operative images and 6‐month post‐operative images.

Typical cases are shown in Figure 4. The surgical procedure required approximately 40 min for each patient. While this surgical technique had been previously performed on over 100 patients, most of them underwent the procedure using modified syringe needles. For this study, we selected 26 individuals who underwent the surgery with the aid of the special guiding needles. The utilization of the guiding needle notably improved the ease of the suture threading process. Among the 26 participants, there were 23 females and 3 males, with an average age of 28.6 years (ranging from 21 to 39 years). All patients reported satisfactory facial outcomes following the surgery and were committed to their 6‐month follow‐up appointments.

### 3.2 Patient Self‐Assessment of the Surgery

Two self‐assessment standards for patients were utilized. The first was the level of satisfaction regarding the postoperative results of the procedure. The score of satisfaction ranged from 1 to 6, with 1 being poor and 6 being very good. The average score for the postoperative effect was 5.692 ± 0.618. Twenty patients gave the operation a “6”, while four patients submitted “5” and two patients submitted “4”. No patient gave scores from 1 to 3. The other self‐assessment related to the patient's surgery tolerance. Given that patients exclusively opted for local anesthesia, assessing their tolerance during surgery was a crucial aspect. Out of the 26 patients, 23 rated their surgical experience as “Very good” while three patients found it to be “Tolerable” Notably, none of the patients rated the experience as “Poor”.

### 3.3 Postoperative Complications

Several complications were taken into consideration: (1) Hematoma: Two patients experienced mild bruising around their lower eyelids, but all bruises resolved within 3 weeks. (2) Infections: There were no instances of infection among the patients. (3) Ectropion: None of the patients exhibited ectropion. (4) Bilateral asymmetry: One patient displayed slight lower eyelid asymmetry, and no one deemed it necessary to undergo a second plastic surgery. (5) Diplopia: Two patients briefly experienced slight diplopia, but this symptom disappeared within 1 week. (6) Orbital Hemorrhage: None of the patients exhibited. (7) Vision loss: None of the patients exhibited.

## 4 Discussion

The transconjunctival approach is a widely favored lower eyelid blepharoplasty procedure in which excess fat pads are removed and repositioned to rectify tear trough deformities. This method is highly regarded for its convenience, safety, and the rapid recovery it affords patients, as it addresses both bulging and depressed deformities simultaneously [1, 2, 9]. The traditional transconjunctival technique conservatively excises lateral and central fat pads following medial dissection and transection of the arcus marginalis; subperiosteal dissection is then performed for the transposition of the fat pads as random‐pattern flaps. The transposed fat pads are fixed with 6‐0 sutures on the outer surface of the skin. The Kawamoto and Bradley technique is also considered a transconjunctival surgery, called “TROUF” that is, transconjunctival repositioning of unpedicled orbital fat [10]. It is similar to the Goldberg technique but differs in that it is performed beneath the tear trough, with the free fat secured internally by 5‐0 sutures. The Hidalgo technique is also like the Goldberg technique but does not involve the extraction of extensive pedicles of fat. Nevertheless, both methods necessitate the use of sutures on the skin's surface to secure the fat flap. In the Freeman technique, surgeons opt to elevate the fat pertaining to the suborbicularis oculi rather than executing orbital fat transposition to correct the tear trough, owing to the theory that the tear trough results from the descent of the malar fat pad and deficiency of suborbicularis oculi fat tissue at the arcus marginalis [11]. Wong and Mendelson applied their extended transconjunctival approach by utilizing the midcheek soft‐tissue spaces and precisely releasing key retaining ligaments to address infraorbital fat herniation and correct the tired appearance [10]. Other techniques exist that use free orbital fat grafts or tipped fat, which also require fixation to the skin surface. However, free fat grafts have a lower survival rate, and fixation to the skin surface increases the risk of infection and need for additional care steps. Internal fixation of tipped fat could address these limitations and enhance the use of orbital fat. Buried guiding needle fixation allows for internal and non‐superficial manipulation, effectively reducing complications such as fat displacement, and we have explored the use of needles for this purpose despite some operational challenges. To design guiding needles suitable for different facial structures, the length and radius of the needle were varied to enhance practicality and convenience during operation and avoid injury of periorbital vessels and nerves.

## 5 Conclusion

This study employed a special guiding needle for transposing the orbital fat pad into the tear trough groove, treating it as a random‐pattern flap, and then internally suturing it to enhance the tear trough appearance. The key aspect of this novel technique involved securely fixing an ample amount of fat tissue to the deep tissue through a single, small transconjunctival incision.

While this modified procedure is based on the transconjunctival approach, it does share certain inherent limitations of the standard transconjunctival method. Notably, it may not fully address the dark pigmentation often associated with tear trough deformity. Therefore, additional therapeutic measures such as laser treatments and fat injections may be required to effectively address this issue [11]. Furthermore, as this procedure does not inherently tighten the skin, it may be necessary to consider supplementary treatments to achieve skin tightening. In summary, this study established a novel and promising technique that is clinically practical and effective for transconjunctival lower eyelid blepharoplasty, incorporating orbital fat repositioning to correct tear trough deformities.

## Author Contributions

Conceptualization: Muran Zhou and Aimei Zhong. Methodology: Jialong Chen, Diandian Li. Writing – original draft preparation: Ke Wu, Jinfei Hou. Writing – review and editing: Ke Wu, Jialong Chen. Supervision: Jiaming Sun. All authors have read and agreed to the published version of the manuscript.

## Ethics Statement

Number [2019] IEC (S864) was approved by the Medical Ethics Committee of Huazhong University of Science and Technology for the studies involving human tissue. This study was conducted in accordance with the declaration of Helsinki. All participants provided informed consent.

## Conflicts of Interest

The authors declare no conflicts of interest.

## Data Availability

The datasets generated and analyzed during the current study are available from the corresponding author on reasonable request.
